# Modified total humeral replacement on unusual osteosarcoma of the humerus: A case report

**DOI:** 10.1016/j.ijscr.2019.04.027

**Published:** 2019-04-17

**Authors:** Yogi Prabowo, Adisa Yusuf Reksoprodjo

**Affiliations:** Department of Orthopaedic & Traumatology, Cipto Mangunkusumo National Central Hospital and Faculty of Medicine, Universitas Indonesia, Jalan Diponegoro No. 71, Central Jakarta, Jakarta 10430, Indonesia

**Keywords:** Osteosarcoma, Limb salvage, Case report

## Abstract

**Introduction:**

Osteosarcoma of the humerus is an unusual case. In order to salvage the limb, resection followed with total humeral replacement is preferred. In situations where those prostheses are not available, a technique of modified total humeral replacement could be used.

**Case presentation:**

A 20-year-old female patient came with osteosarcoma of the right humerus accompanied by pathological humeral shaft fracture, with the onset of six months. The lesion extended along the proximal to distal part of humerus. The patient underwent a neoadjuvant chemotherapy before surgery and a limb salvage surgery by wide excision of the humerus and reconstruction using modified total humeral replacement was performed. Modifications to the shoulder hemiarthoplasty and total elbow prostheses were made, so both prostheses could join as one unit.

**Discussion:**

After the surgery, no disturbances of hand function were seen, and functional outcome was evaluated by using Musculoskeletal Tumour Society Score (MSTS) score with score of 83% (excellent). After 3 years of follow up, patient could work and resume daily activity with improved MSTS score. There were neither sign of tumor recurrence nor distant metastasis on follow up.

**Conclusion:**

Total Humeral Replacement for the treatment of humerus malignancy was feasible by using this modification. This procedure yielded good functional outcome.

## Highlights

• The patient underwent a neoadjuvant chemotherapy before surgery, but the mass became more expanded.
• Limb salvage surgery by wide excision of the humerus and reconstruction using modified total humeral replacement was done.
• Total Humeral Replacement for the treatment of humerus malignancy was feasible by using this modification.
• This procedure yielded good functional outcome.

## 1 Introduction

The humerus is commonly affected by osteosarcoma, especially the proximal humerus, which ranks third in the osteosarcoma predilection sites [1]. However, osteosarcoma lesion which appeared in the whole humerus is a rare finding. We reported a patient who came to seek medical treatment after the emergence of a pathological fracture on her right upper arm. Ùpon physical and radiological examination, we found an osteogenic tumor involving her right humerus. We assessed the motor, sensory, and motoric function of the upper extremity. Her hand functioned properly except for a limited movement in elbow and shoulder.

Total humeral osteosarcoma remains a challenge for treatment. Currently, osteosarcoma of the humerus has been successfully treated with limb salvage procedure with modular prosthesis as the preferred implant [2]. Limb salvage surgery offers a better functional outcome with no difference in survival rate of the patient compared to ablative surgery.

Unfortunately, the modular prosthesis for total humeral is not available in our place of practice [3]. Therefore, we modified the available prostheses to perform total humeral replacement. We performed a wide excision and combined a shoulder hemiarthroplasty and a total elbow prosthesis to preserve the upper arm. Post-operatively, the patient retained her sensory and motoric function, with no complaints after three years of follow up. This case report was made according to the SCARE guidelines [4].

## 2 Case presentation

A 20-year-old female presented to our center with a telangiectatic osteosarcoma of the humerus. There was no history of malignancies within the family.

**Fig. 1:**
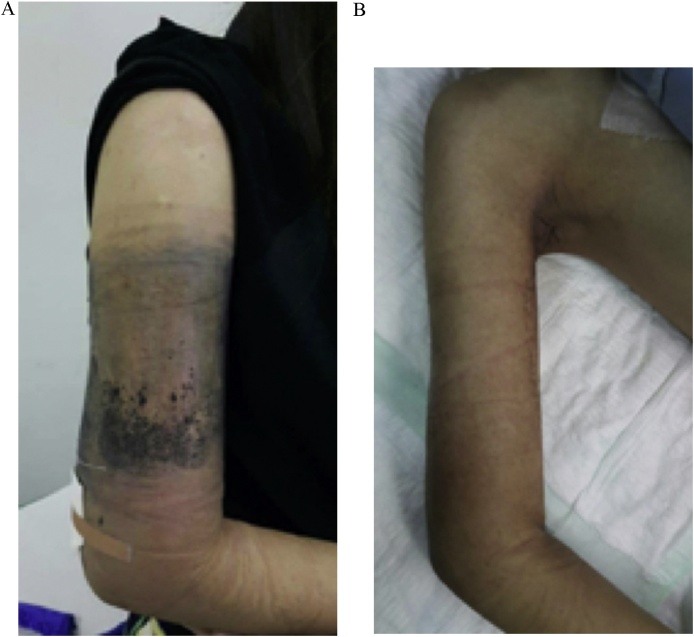
Preoperative Clinical State. A. Initial state, B. Post neoadjuvant chemotherapy.

MRI examination and open biopsy were performed by the previous physician. At the time the results were thought to be malignant lymphoma. After several diagnostic trials, the patient visited our center to seek advice about her telangiectatic osteosarcoma (Fig. 1A).

**Fig. 2:**
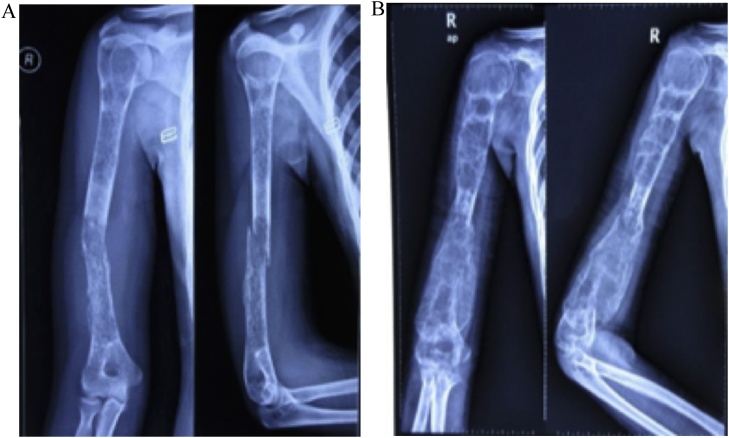
Anteroposterior and Lateral Radiograph of Humerus. A. Initial state, B. Post neoadjuvant chemotherapy.

**Fig. 3:**
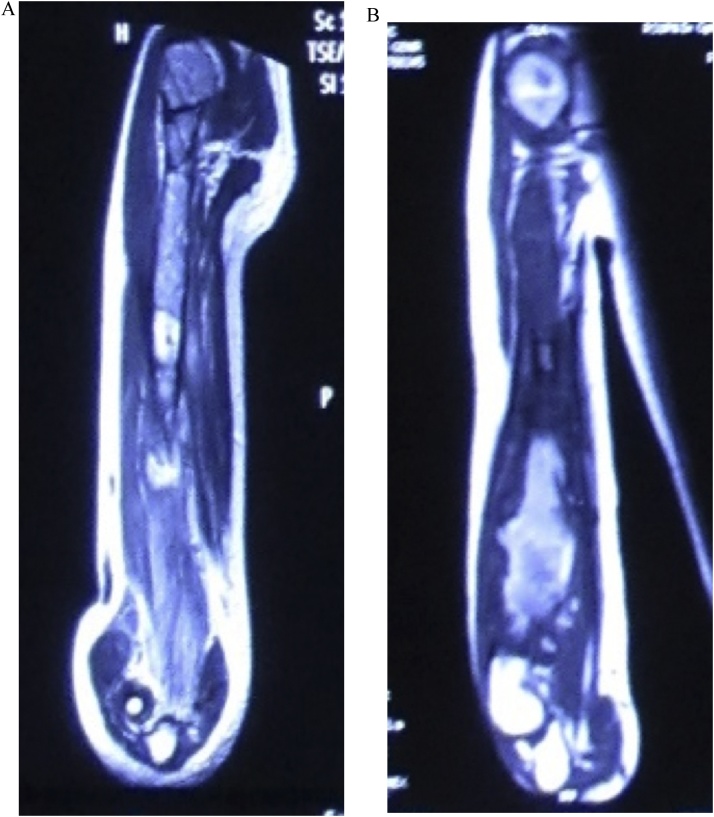
MRI of the humerus. A. Initial state, B. Post neoadjuvant chemotherapy.

We observed a circumferential mass on the distal part of the right arm with a slight deformity of the arm, with marked venous engorgmnt and distal edema. The mass was warm and solid on palpation. Function of the right hand was still preserved. From the laboratory findings, there were marked elevation of the alkaline phosphatase and lactate dehydrogenase. From humerus X-ray, there was mixed lesions along the humerus with pathological fracture on the midshaft (Fig. 2A). T2-weighted MRI showed *iso*-hyperintense and expansile lesions along the humerus (Fig. 3A). For the metastatic workup, chest X-ray showed no coin lesions or metastatic characteristics. Patient also had a PET scan and the result was unremarkable. From the Clinico Pathological Conference (CPC) forum, it was concluded that the diagnosis was osteosarcoma of the right humerus stage IIB according to the Enneking classification.

Patient took neoadjuvant chemotherapy regimen with Doxorubicin and Cisplatin for three cycles from January to March 2016. At the end of the third cycles, clinical and radiological evaluations were performed. Clinically the mass was not getting bigger compared to before chemotherapy (Fig. 1B). From x-ray, the mixed lesions became more marked compared to the previous x-ray (Fig. 2B). MRI showed that the mass was slightly became larger compared to the previous MRI with no involvement of neurovascular bundle (Fig. 3B).

**Fig. 4:**
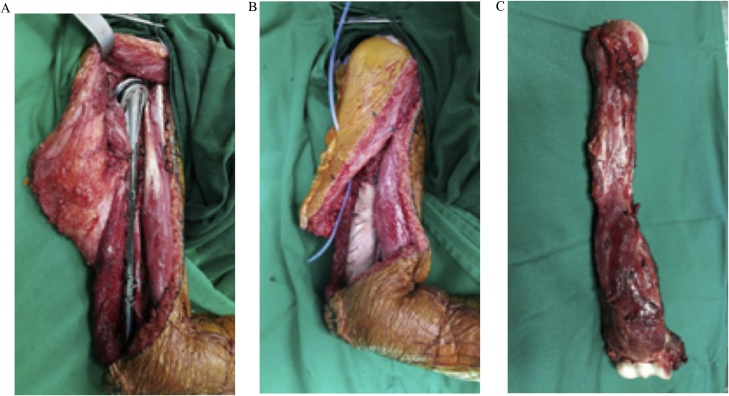
Intraoperative. A. Endoprosthetic assembly, B. Bone Cement and mesh used, C. Resected Humerus.

Six months after the initial complaint, the surgery was performed. We used extensive deltopectoral approach with anterolateral extension through the proximal part and curved backward to complete the posterior distal humerus and elbow approach. This approach was used because of the previous biopsy tract was in the posterior aspect of the distal humerus. The vascularity, rotator cuff tendons, biceps and triceps muscle, and majority of the nerves (musculocutaneous, radial, median, and ulnar nerves) were spared during the total resection of the humerus. Axillary nerve was sacrificed during the tumor resection. To reconstruct the humerus, both long shoulder hemiarthroplasty and total elbow prostheses were used. These two prostheses were joined using two long one-third tubular plate that worked as the long stem augmentation. Extension cerclage wire was used to make the implant as one unit (Fig. 4A). Finally, the stem was augmented with a bone cement from proximal through the distal. Prolene mesh then was sheathed to the bone cement, and then the preserved rotator cuff tendons and biceps and triceps muscle were sutured back with the Ethibond sutures (Fig. 4B). The resected tumor and humerus were then sent to the lab for histopathologic analysis (Fig. 4C).

**Fig. 5:**
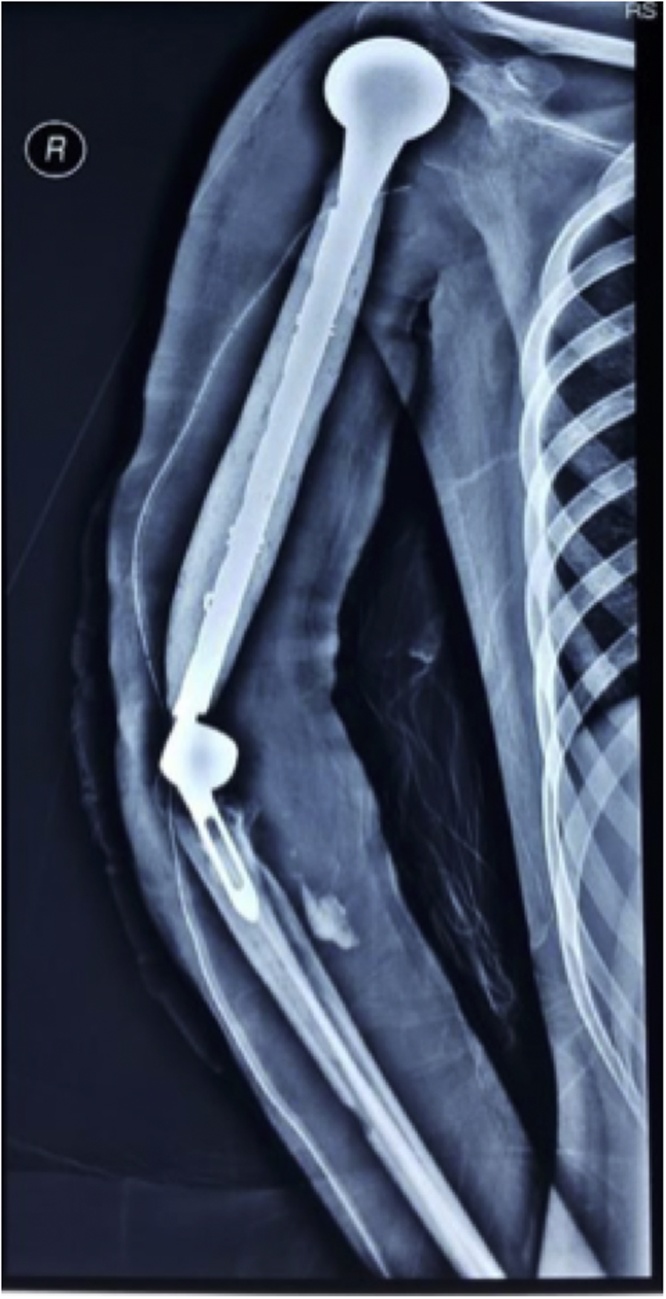
Postoperative radiograph.

**Fig. 6:**
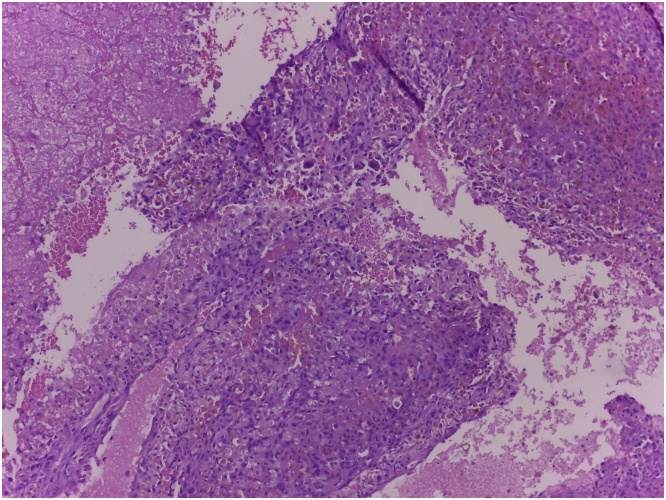
Histopathologic Examination of the Lesion.

**Fig. 7:**
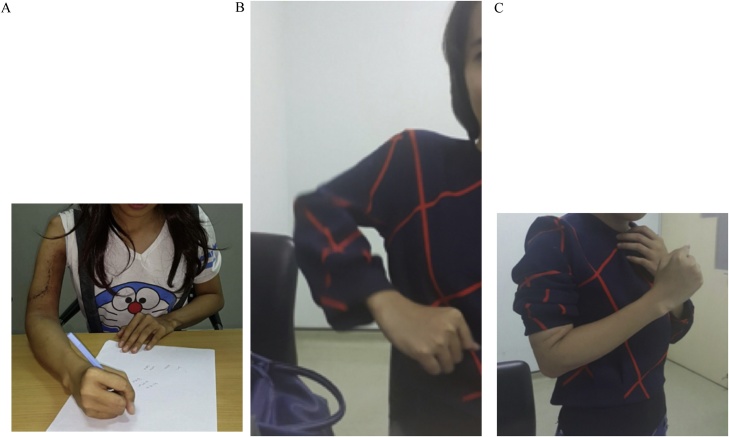
3 Years follow up.

From the post-operative x-ray, the modified prosthesis sat well on the shoulder and elbow joint (Fig. 5). Function of the hand was excellent post-operatively. Patient also could immediately flex her elbow. Post-operative histopathological examination showed telangiectatic osteosarcoma with HUVOS IV (Fig. 6). After the wound healed without complication, patient underwent adjuvant chemotherapy. Two months after the surgery, patient could start writing with her right hand without marked difficulties. Further follow up of three years post-operatively, patient already came back to work and were able to perform daily activities without difficulties. Patient’s shoulder abduction and elbow flexion was shown on the pictures (Fig. 7). The MSTS functional score for the upper limb scored 83% which was excellent. There were no post-operative complications and the immunohistochemical workup used to rule out lymphoma (CD20, CD15, CD 30, and CK) would be planned.

## 3 Discussion

The humerus, especially proximal part, is a common site for the primary osteosarcoma on the upper limb, being the third site to be the most affected after the distal femur and proximal tibia.^1^ Osteosarcoma is mainly on the metaphyseal region of the bone, but in this case, it extended along the humerus, thus made it an unusual variant of osteosarcoma. Not many osteosarcoma of the whole humerus that can be found in other studies regarding total humeral replacement. The second decade of life is the peak incidence of osteosarcoma cases, which matches with our patient. Approximately 58% of osteosarcoma patients are male, it means that females are more often to get an osteosarcoma compared to males [2].

This patient came with a chief complaint of sudden pain on her right arm after she heard a crack sound when she lifted a baby. The patient had no complaint on her arm before. On osteosarcoma cases, pathologic fracture is rare as the chief complaint [1]. Pain, accompanied by a tender and soft tissue swelling is the most common complaint.

From radiological findings, we got the results of the mixed lesions on the x-ray. MRI showed a hyperintense extracompartemental lesions along the humerus. Laboratory findings showed an increased level of alkaline phosphatase and lactate dehydrogenase. These findings were consistent with characteristics of the osteosarcoma [1,2]. The mixed lesions on the x-ray is found on the 25% of the cases. But for the lesion to extend along the length of the humerus is unusual. The probable cause of expanded tumor mass after chemotherapy is that traditional chemotherapy not only kills a fraction of tumor cells, but also activates the stroma and can promote the growth and survival of residual cancer cells to foster tumor recurrence and metastasis.

Limb salvage procedure was chosen for this case with resection of the whole humerus, followed by modified total humeral replacement. Before the surgery we had the patient completed the neoadjuvant chemotherapy regiment for three cycles in three months. Neoadjuvant or induction chemotherapy using multiple drugs, in this case Doxorubicin and Cisplatin without high dose methotrexate, is a modality used to increase the survival rate of limb salvage procedure by preventing the tumor metastases to the lung and reducing the emergence of drug-resistant tumor cells [2]. Chemotherapy also helps in reducing the size of the tumor, although it was not the case in our patient. Chemotherapy helps to confine the tumor within the calcified periosteum, so we can have a better demarcated tumor margin, thus increasing the success rate of the surgery. The response of therapy is classified based on the HUVOS necrosis grading system. This classification system is widely used for evaluation of chemotherapy in osteosarcoma. Later on, the post-operative-histopathological assessment of this lesion was osteosarcoma HUVOS IV, which means complete destruction of the tumor cells.

There is relatively better prognosis of telangiectatic osteosarcoma (TOS) than that of other variants of osteosarcoma. There is important involvement of CEACAM1 in angiogenesis. CEACAM1 is a major effector of vascular endothelial growth factor (VEGF) in early tumor microvessel formation. VEGF increases CEACAM1 expression on both mRNA and protein levels, and the administration of a monoclonal CEACAM1 antibody blocks in vitro VEGF-induced endothelial tube formation. Furthermore, a disease that produces severe local inflammation accompanied by accumulation of CD11b cells at the site of infection, a VEGF-independent role of CEACAM1 has been characterized. Both blood and lymphatic vessel formation appear to be affected by the loss of CEACAM1/CD11b cells, which control angiogenesis during inflammation. Due to its capacity to evade the immune system, as well as its potent proangiogenic effects, CEACAM1 appears to play an important role in tumor growth and progression.

The tumor on this patient extended along the humerus, thus making it an excellent candidate for the total humeral replacement. Total humeral replacement itself is a rare procedure, and so are the studies that published the results of this procedure. All studies tried as they could to preserve the rotator cuff, biceps, deltoid tendons, and also the nerves including axillary, radial, median, and ulnar nerves [3]. For the elbow joint reconstruction they preserved the triceps tendon. In our case, axillary nerve couldn’t be preserved, but other important structure could be preserved as can be seen postoperatively, the hand function was good.

The preferred prostheses following resection of the tumor is the Modular Replacement System (MRS) [2]. At the time of the surgery, the MRS was not available at our practice. So, we performed the modified total humeral replacement. Several studies talked about a custom-made prosthesis, but its usage is for the total humeral replacement done on the paediatric patient, which is to accommodate the bone growth [[5], [6], [7]]. Thus making our modified total humeral replacement the first case to utilize such approach to the conventional total humeral replacement.

Fabroni et al. used a custom-made endoprosthetic for adult and had long-term outcomes from his three cases of total humerus replacement [8,9]. The difference between Fabroni and our case is that Fabroni self-produced the total humeral prostheses, while we used the available prostheses on the market and assembled it into the whole humerus. To our knowledge, this kind of modified total humeral prostheses have not been done elsewhere.

It is cheaper compared with the MRS and easily available. Our concern is the longevity of the prostheses, because it was assembled using a separated component with plate, wire, and bone cement. The construction was firm during the operation, but longer term of follow up is needed to evaluate the results.

Post operatively, patient had a functional-scores of 83%, based on the MSTS for the upper limb. This finding corresponded to other studies who had average scores of 71.5% [10]. Follow up had been done for three years post operatively, and the results was sexcellent. Other studies average follow up is 42.9 months [11]. Based on their study, infection is one of the main problem that caused the total humeral replacement to be failed. Fortunately, patient exhibit no such signs nor symptoms.

Modified total humeral replacement was performed for the unusual osteosarcoma of right humerus in twenty-year-old female with excellent post operative hand function. With long term follow up and good documentation, this modification could serve as cheaper alternative for MRS with good amount of availability and flexibility.

## Conflict of interest

The authors declare that there are no conflicts of interest regarding the publication of this article.

## Sources of funding

The authors declare that the study sponsors had no involvement in the collection, analysis and interpretation of data, in the writing of the manuscript, and in the decision to submit the manuscript for publication.

## Ethical approval

The study has been approved by institution’s ethical committee.

## Consent

Written informed consent was obtained from the patient for publication of this case report and accompanying images.

## Author’s contribution

Adisa Yusuf Reksoprodjo and Yogi Prabowo performed the treatment, performed measurement, and wrote the manuscript. Rizky Priambodo Wisnubaroto helped wrote the manuscript.

## Registration of research studies

N/A.

## Guarantor

Yogi Prabowo.

## Provenance and peer review

Not commissioned, externally peer-reviewed.
